# Correction to: Transcriptome analyses of tumor-adjacent somatic tissues reveal genes co-expressed with transposable elements

**DOI:** 10.1186/s13100-019-0183-2

**Published:** 2019-10-10

**Authors:** Nicky Chung, G. M. Jonaid, Sophia Quinton, Austin Ross, Corinne E. Sexton, Adrian Alberto, Cody Clymer, Daphnie Churchill, Omar Navarro Leija, Mira V. Han

**Affiliations:** 1 School of Life Sciences, University of Nevada, Las Vegas, NV 89154 USA; 2 Department of Computer Science, University of Nevada, Las Vegas, NV 89154 USA; 3 Nevada Institute of Personalized Medicine, Las Vegas, NV 89154 USA

**Correction to: Mobile DNA**

**https://doi.org/10.1186/s13100-019-0180-5**

Following publication of the original article [1], the authors reported errors in Table 2 wherein all “KZFP” in the gene names should be changed to “ZNF”.

The correct version of the table is provided here.

**Table 2:** KZFP gene members in core TE modules. a. KZFP genes that are members of the core TE modules. b. KZFP genes in module M3. M3 is in high correlation with the core TE modules (Supplementary Figure 5 a. and b.)

a.		
core TE modules	KZFP	chromosome
M8	*HKR1*	19
	*ZNF226*	19
	*ZNF682*	19
	*ZNF789*	7
	*ZNF814*	19
M21	*ZNF404*	19
	*ZNF418*	19
	*ZNF589*	3
	*ZNF75A*	19
M38	*ZNF117*	7
M45	*ZNF334*	20
	*ZNF493*	19
	*ZNF506*	19
	*ZNF721*	4
	*ZNF737*	19
b.		
KZFPs in module M3	chromosome	
*ZNF169*	9	
*ZNF202*	11	
*ZNF266*	19	
*ZNF300*	5	
*ZNF320*	19	
*ZNF431*	19	
*ZNF439*	19	
*ZNF44*	19	
*ZNF587*	19	
*ZNF662*	3	
*ZNF7*	8	
*ZNF700*	19	
*ZNF708*	19	
*ZNF714*	19	
*ZNF732*	4	
*ZNF83*	19	
*ZNF841*	19	
