# Supplementary material for: Transcriptome analyses of tumor-adjacent somatic tissues reveal genes co-expressed with transposable elements
Source: Mob DNA. 2019 Sep 3;10:39. doi: 10.1186/s13100-019-0180-5 (PMC6720085; doi:10.1186/s13100-019-0180-5)

# **Additional** **Files**

## Supplementary Table 1. Tissue types and the number of normal tissue samples

RNA-seq data of non-tumorous samples were collected from the Cancer Genome Atlas. BLCA (Bladder urothelial carcinoma) , BRCA (Breast carcinoma), COAD (Colon adenocarcinoma), ESCA (Esophageal adenocarcinoma), HNSC (Head and neck squamous cell carcinoma), KICH (kidney chromophobe ), KIRC (kidney renal clear cell carcinoma ), KIRP (Kidney renal papillary cell carcinoma ), LIHC (Liver hepatocellular carcinoma), LUAD (Lung adenocarcinoma), LUSC (Lung squamous cell carcinoma), PRAD (Prostate adenocarcinoma), READ (Rectum adenocarcinoma), STAD (Stomach adenocarcinoma), THCA (Thyroid carcinoma) and UCEC (Uterine Corpus Endometrial Carcinoma)

| tissue | samples |
| --- | --- |
| BLCA | 19 |
| BRCA | 113 |
| COAD | 41 |
| ESCA | 11 |
| HNSC | 44 |
| KICH | 25 |
| KIRC | 72 |
| KIRP | 32 |
| LIHC | 50 |
| LUAD | 59 |
| LUSC | 51 |
| PRAD | 52 |
| READ | 10 |
| STAD | 35 |
| THCA | 59 |
| UCEC | 24 |
| Total | 697 |

## Supplementary Table 2. Tissue clustering results based on TE expression

Normalized Mutual Information values for tissue clustering, based on different classes of transposable element expression.

|  | TE family | TE locus 100K away from genes | TE locus 1K away from genes | TE locus |
| --- | --- | --- | --- | --- |
| random | 3.13E-04 | 2.19E-04 | 2.75E-04 | 2.38E-04 |
| young L1 |  | 0.209 | 0.381 | 0.189 |
| HERV |  | 0.639 | 0.867 | 0.895 |
|  |  |  |  |  |
| LTR | 0.633 | 0.887 | 0.907 | 0.890 |
| DNA | 0.326 | 0.852 | 0.887 | 0.929 |
| SINE | 0.238 | 0.742 | 0.892 | 0.851 |
| LINE | 0.275 | 0.890 | 0.927 | 0.950 |
| gene | 0.955 | 0.962 | 0.935 | 0.935 |


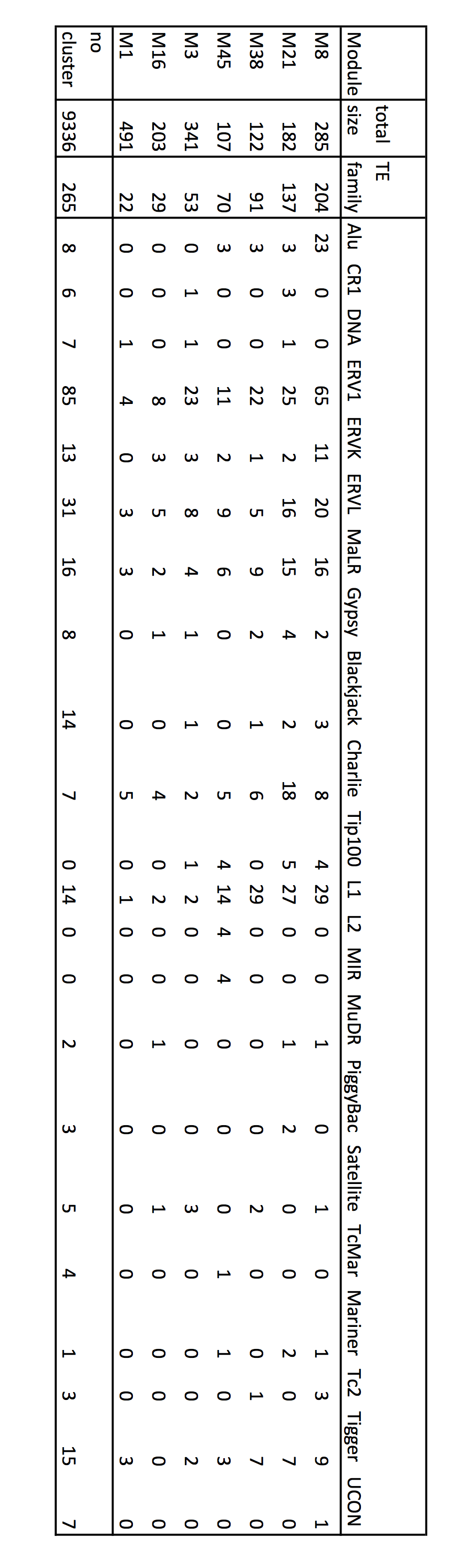


## Supplementary Table 3. TE modules and TE family memberships

TE co-oexpression modules and the TE families belonging to each TE module. We consider the top four modules, M8, M21, M38, M45 with the largest TE family membership as core TE modules. M3 is not defined as a TE module, since it has more gene members than TE family members, but it showed strong correlation with all four TE modules.

And the TE family distribution

## Supplementary Table 4. KZFP members of the intronic TE module

KZFP members belonging to the intronic TE module N1, and module N10 that is correlated with module N1.

| KZFP members in the intronic TE module N1 | chromosome | KZFP members in correlated module N10 | chromosome |
| --- | --- | --- | --- |
| PRDM7\|11105 | 16 | ZNF44\|51710 | 19 |
| ZNF169\|169841 | 9 | ZNF738\|148203 | 19 |
| ZNF226\|7769 | 19 | ZNF439\|90594 | 19 |
| ZNF266\|10781 | 19 | ZNF337\|26152 | 20 |
| ZNF26\|7574 | 12 | ZNF334\|55713 | 20 |
| ZNF354B\|117608 | 5 | ZNF662\|389114 | 3 |
| ZNF700\|90592 | 19 | ZNF83\|55769 | 19 |
| ZNF7\|7553 | 8 | ZNF493\|284443 | 19 |
| ZNF789\|285989 | 7 | ZNF211\|10520 | 19 |
| ZNF814\|730051 | 19 | ZNF682\|91120 | 19 |
| ZNF841\|284371 | 19 |  |  |

## Supplementary Table 5. Gene correlated with L1HS 5’ transcript level

The list of genes correlated with L1HS 5’ transcript level in more than one tissue. Gene names, the tissue where the significant correlation was found, coefficient of the gene estimated from the best linear model, p-value for the gene coefficient, q-value, and partial eta-squared for the gene are reported.

## Supplementary Table 6. Housekeeping genes

List of housekeeping genes plotted in Figure 5 as reference.

| Caracausi et al. 2017 [1] | Eisenberg et al. 2013 [2] |
| --- | --- |
| ACTG1  RPS18  POM121C  MRPL18  TOMM5  YTHDF1  TPT1  RPS27 | C1orf43  CHMP2A  C15orf24  EMC7  GPI  PSMB2  PSMB4  RAB7A  REEP5  SNRPD3  VCP  VPS29 |

1. Caracausi M, Piovesan A, Antonaros F, Strippoli P, Vitale L, Pelleri MC. Systematic identification of human housekeeping genes possibly useful as references in gene expression studies. Mol Med Rep. 2017;16:2397–410.

2. Eisenberg E, Levanon EY. Human housekeeping genes, revisited. Trends Genet TIG. 2013;29:569–74.

## Supplementary Figure 1. Effect of normalization on TE transcripts

Total TE derived transcript count before and after normalization. Left panel shows the total reads counted by TEtranscripts plotted against the library size (total reads in the fastq file). Right panel shows the normalized read counts after the normalization process described in the Methods, again plotted against the library size.

## Supplementary Figure 2. Example cases of correction for intron retention.

Cases where intron retention leads to TE derived transcripts. From top to bottom, AluSx1_dup59209(chrY:21153222-21153521), L2a_dup21781(chr2:113980079-113981081), L1MA7_dup4297 (chr8:134015602-134015763). AluSx1 is embedded within an exon of gene *TTY14*, L2a is embedded in an intron of *PAX8*, L1MA7 is embedded in an intron of gene *TG*. In all three cases, read counts for the focal TEs were reduced to zero, and the reads mapping to these TEs did not contribute to the overall TE family count.

## Supplementary Figure 3. Comparison of TE family expression between multi-mapped reads and uniquely mapped reads.

Family level TE transcript quantification based on uniquely mapped reads (Bowtie1) and multi-mapped read(STAR) for six different TE families. AluSx1, AluYa5, HERVK3-int, HERVK9-int, L1HS and LTR5_Hs.

## Supplementary Figure 4. Read alignment for TEs with tissue-specific expression

RNA-seq read alignments for four out of the sixteen TEs with tissue-specific expression shown in Figure 4 are visualized using IGV. On top of the reads is the track showing the 48bp mappability. On the bottom are the RefSeq gene annotations and the TE annotations from Repeatmasker. a. HERVIP10F-int_dup69 for sample TCGA-K4-A54R (BLCA) b. L1MA1_dup4121 and L2a_dup164397 for sample TCGA-BH-A0AZ (BRCA) c. ERV3-16A3_I-int_dup4366 for sample TCGA-AZ-6599 (COAD) d. L1MA3_dup4399 for sample TCGA-FL-A1YV (UCEC).

## Supplementary Figure 5. co-expression modules in the weighted gene co-expression network analysis

Co-expression modules identified through the weighted gene co-expression network analysis (WGCNA) is visualized for the breast tissue data. The four TE modules M8, M21, M38, M45 are marked with a red *. a) Relationship between the 61 modules. b) correlation between the 61 modules. c) topological adjacency matrix used to identify the 61 modules.

## Supplementary Figure 6. Transcription factor binding on KZFP genes that are members of the intronic TE module N1.

a) Transcription Factor binding enriched upstream of the genes that members of the intronic TE module N1. b) transcription factors enriched and the cell line that was assayed. Transcription factor binding enrichment is calculated by the EnrichR platform, based on the ChIP-Seq data collected in the ENCODE and ChEA databases.

## Supplementary Figure 7. Transcription factor binding on genes correlated with L1HS 5’

Transcription Factor binding enriched upstream of the genes that are positively correlated with L1HS 5’ transcript level. Transcription factor binding enrichment is calculated by the EnrichR platform, based on the ChIP-Seq data collected in the ENCODE and ChEA databases.

## Supplementary Figure 8. past radiation therapy and intronic TE expression

a. Intronic TE module expression profile. and b. total normalized read counts of all gene and TE members in intronic TE module N1. Plotted against the past radiation therapy in thyroid tissue.

## Supplementary Figure 9. ENCODE candidate regulatory element marks overlapped with TE expression and ZFP binding

a-b. Overlap of TE expression and KZFP binding with ENCODE Candidate Regulatory Element marks. c-d. Proportion of each category of TEs that are marked with ENCODE Candidate Regulatory Element marks. a. and c. are TE counts in gene regions (including introns and +- 1Kb of start and end of genes). b. and d. are TE counts in intergenic regions (+-1Kb away from start and end of genes).


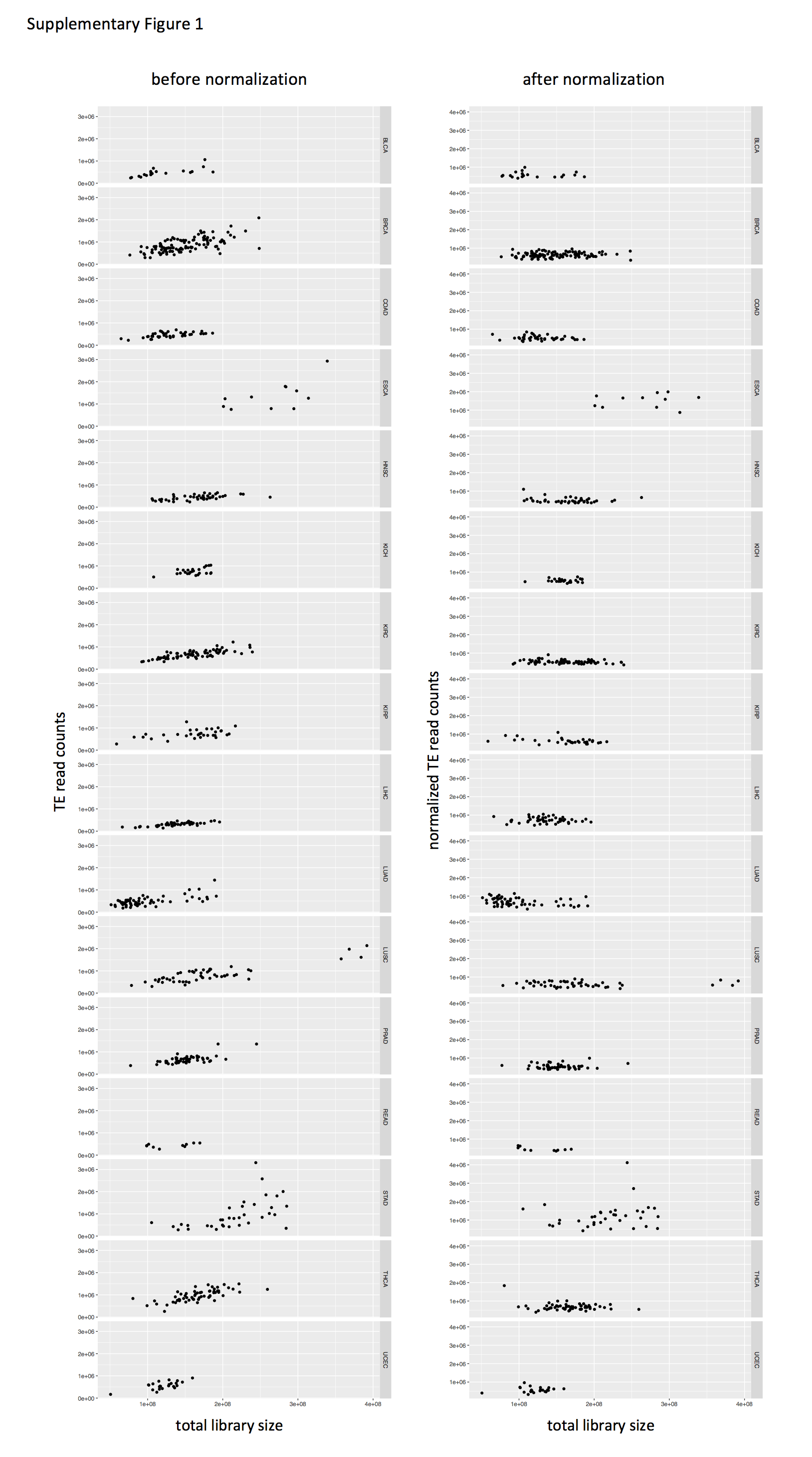


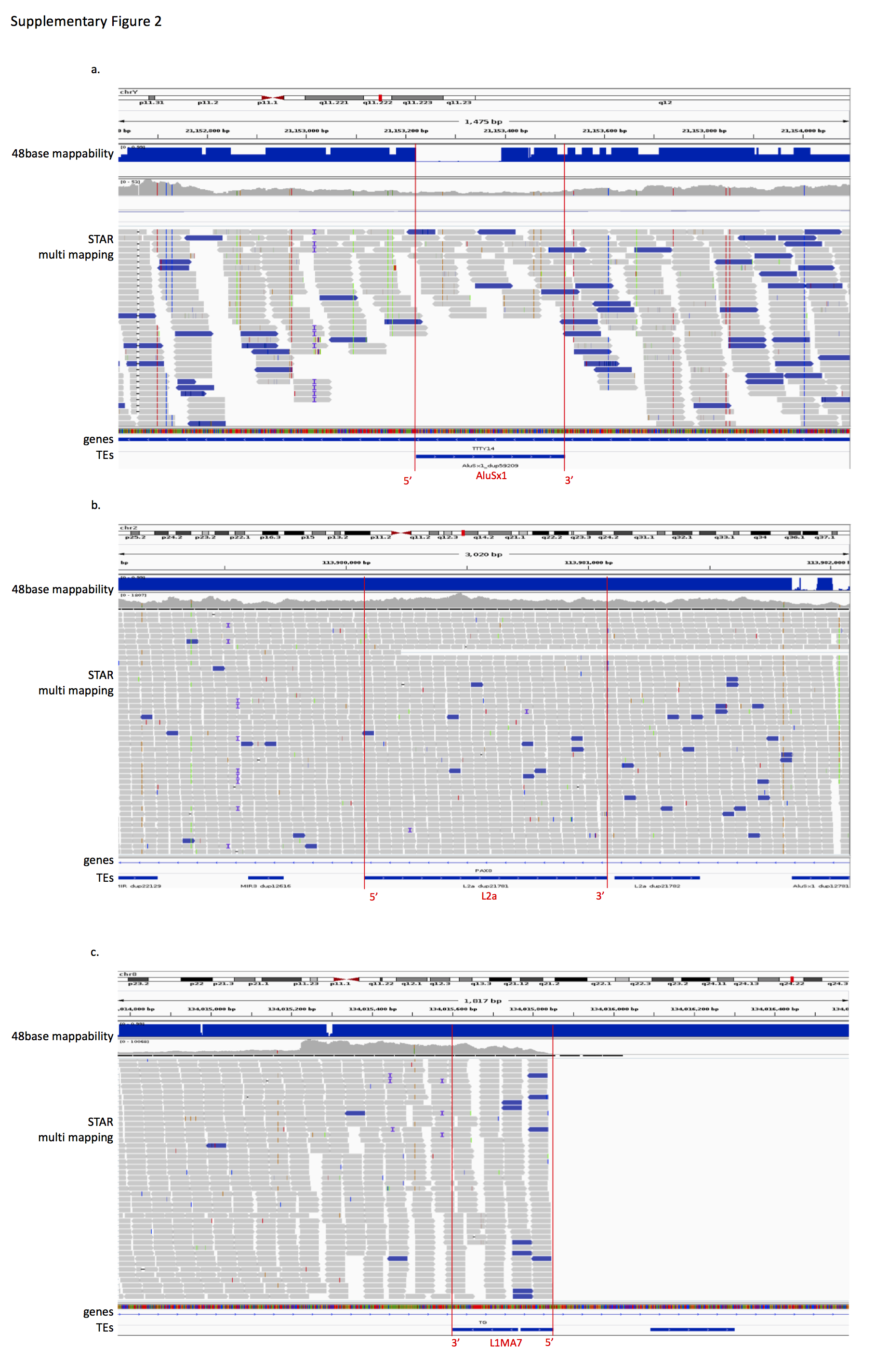


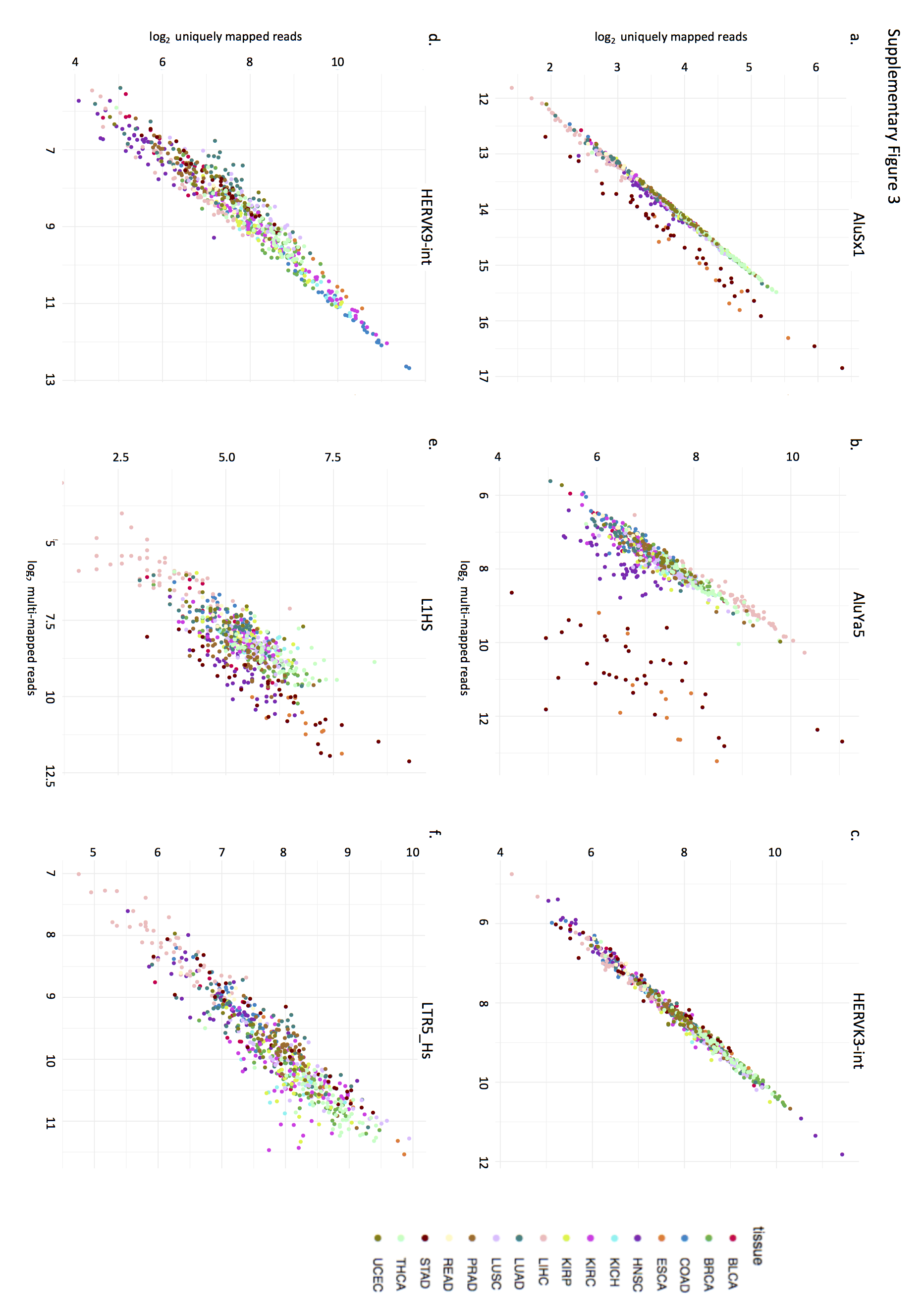


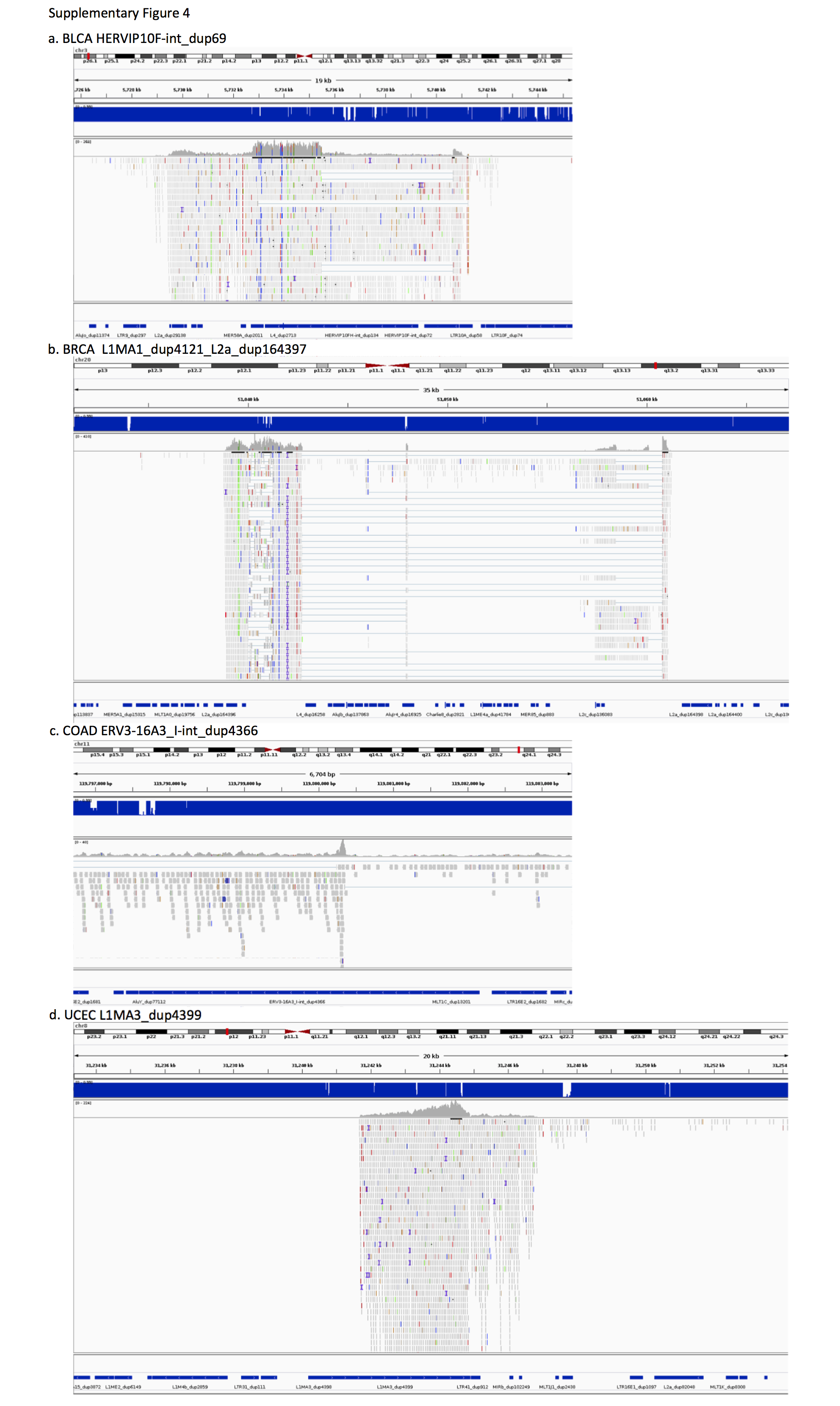


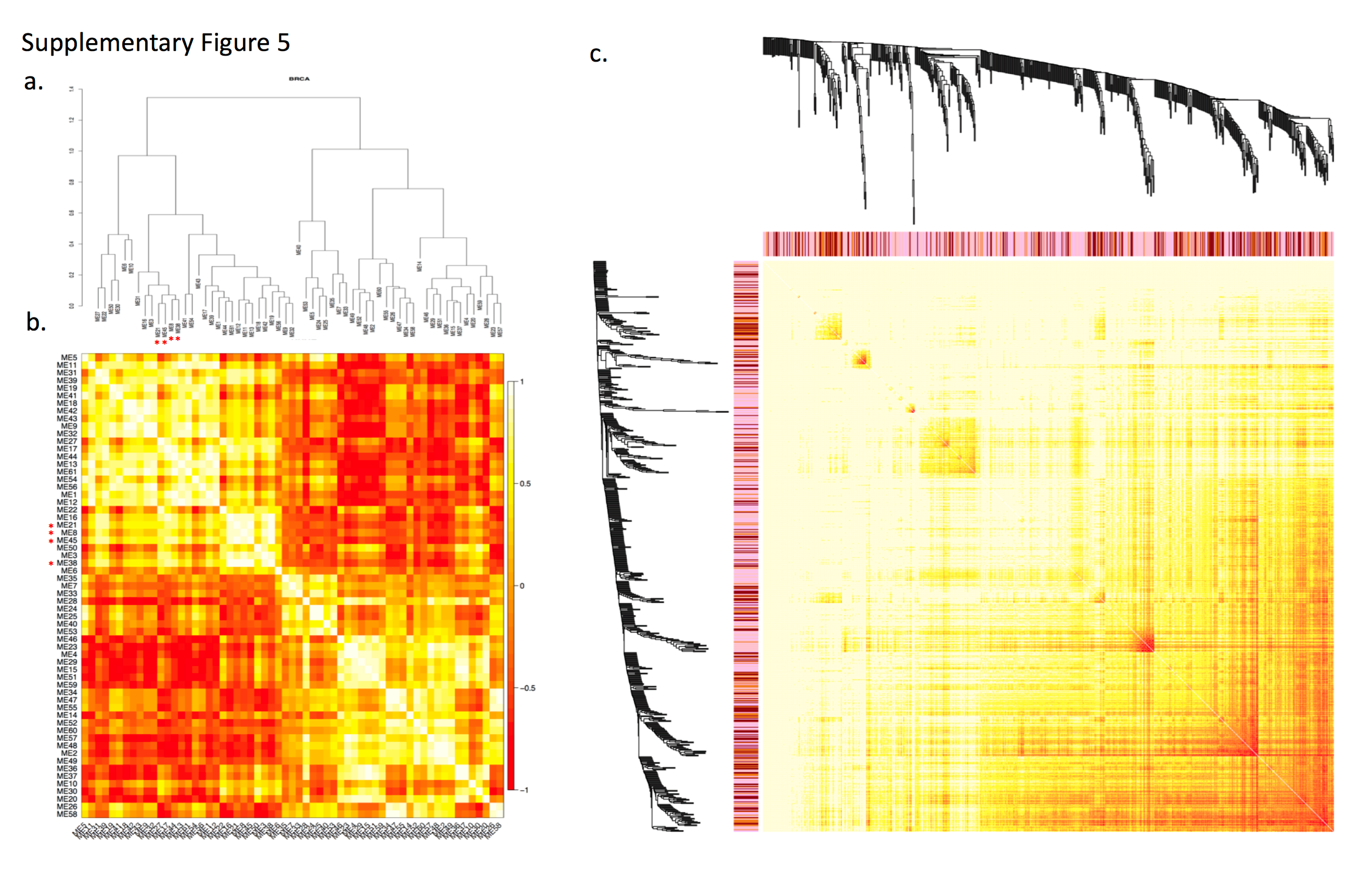

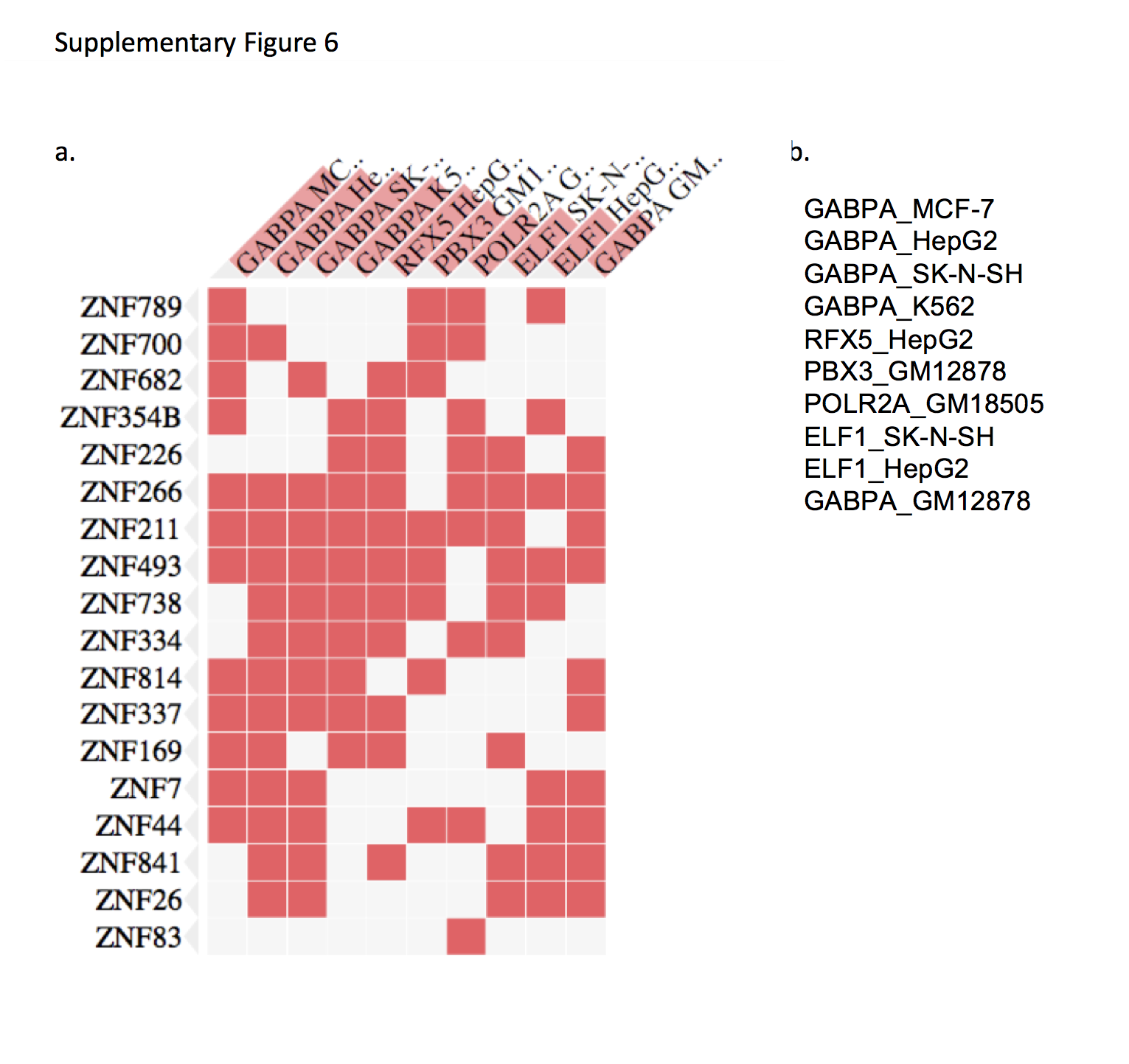

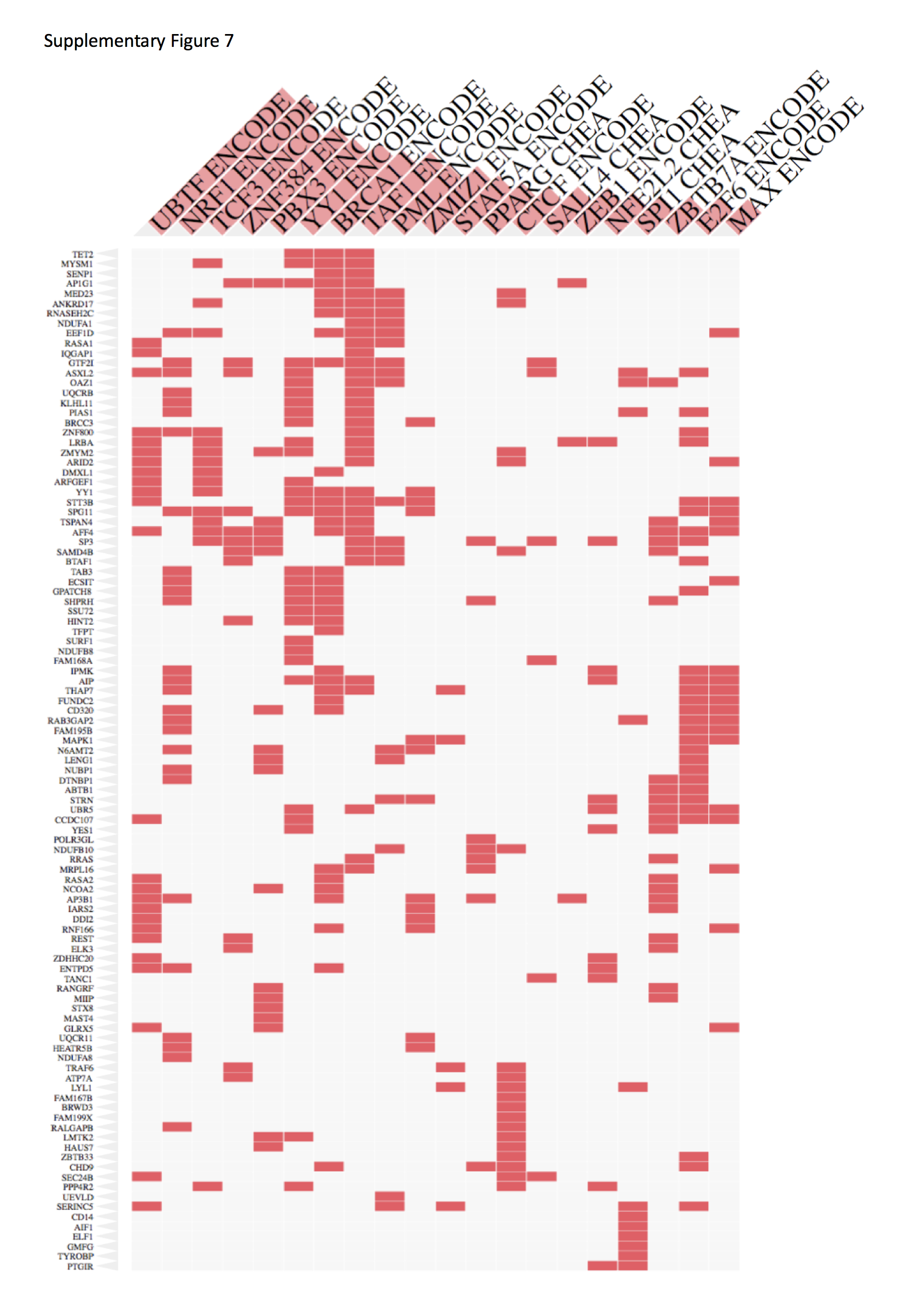

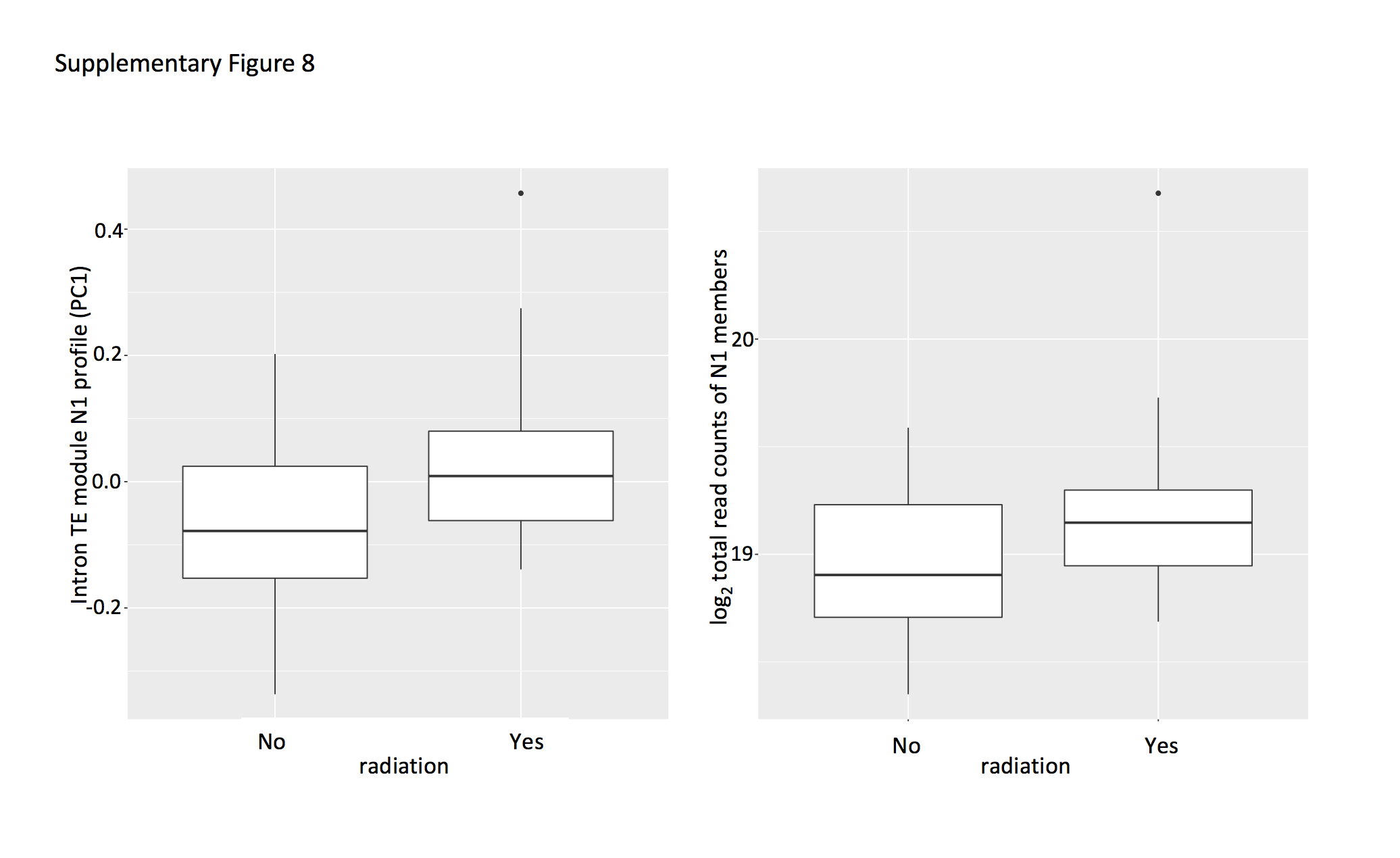


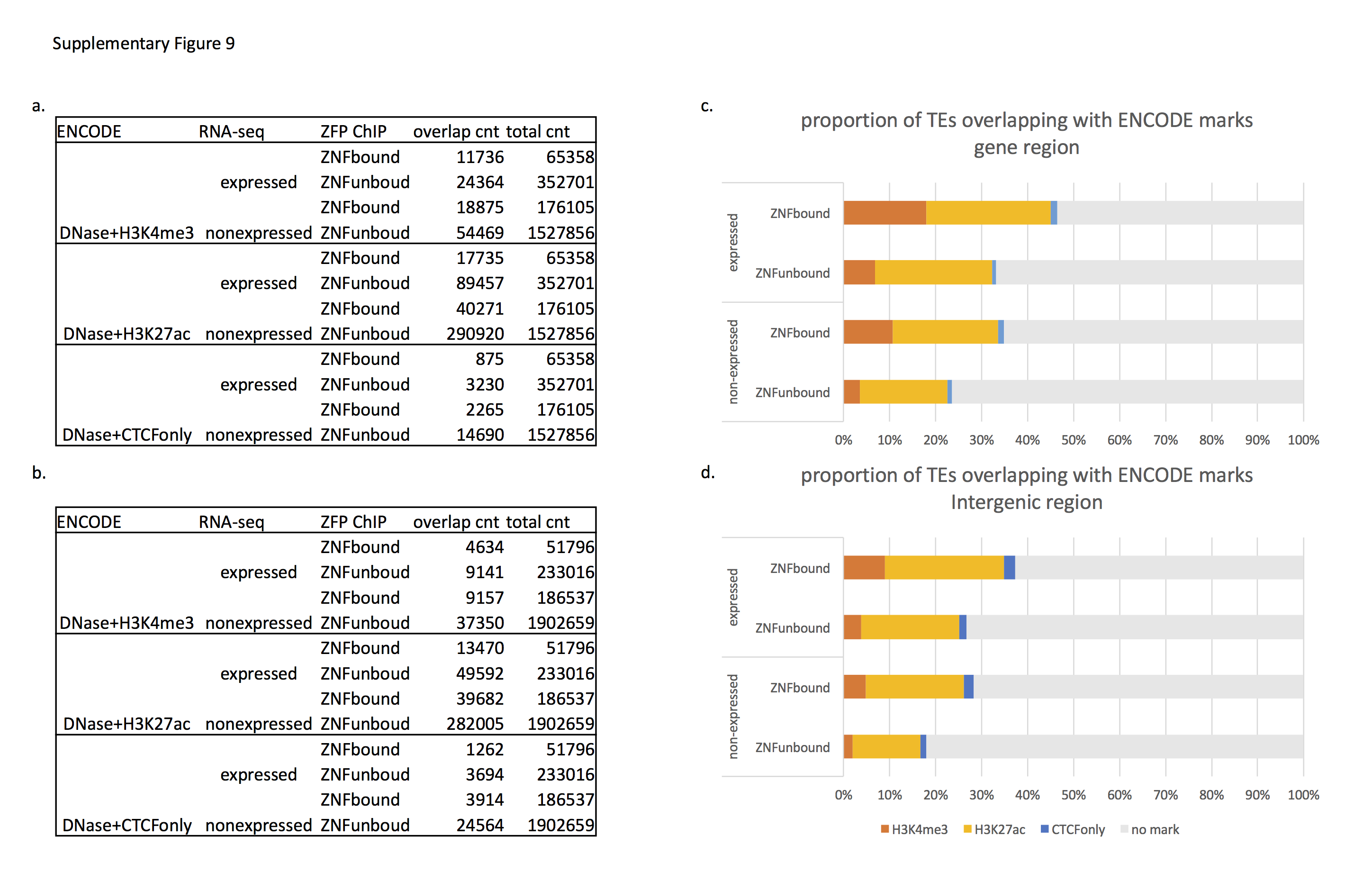

Supplement: Supplementary file 1 — Table S1. Tissue types and the number of normal tissue samples. Table S2. Tissue clustering results based on TE expression. Table S3. TE modules and TE family memberships. Table S4. KZFP members of the intronic TE module. Table S5. Gene correlated with L1HS 5′ transcript level. Table S6. Housekeeping genes. Figure S1. Effect of normalization on TE transcripts. Figure S2. Example cases of correction for intron retention. Figure S3. Comparison of TE family expression between multimapped reads and uniquely mapped reads. Figure S4. Read alignment for TEs with tissue-specific expression. Figure S5. co-expression modules in the weighted gene coexpression network analysis. Figure S6. Transcription factor binding on KZFP genes that are members of the intronic TE module N1. Figure S7. Transcription factor binding on genes correlated with L1HS 5′. Figure S8. past radiation therapy and intronic TE expression. Figure S9. ENCODE candidate regulatory element marks overlapped with TE expression and ZFP binding. (DOCX 8440 kb) [file 13100_2019_180_MOESM1_ESM.docx]
